# Boosting the antioxidant power of *Palmaria palmata* using hydrogen peroxide

**DOI:** 10.1038/s41598-025-03785-z

**Published:** 2025-06-05

**Authors:** Pierre Liboureau, Daniela Maria Pampanin

**Affiliations:** Department of Chemistry, Bioscience and Environmental Engineering, University of Stavanger, Stavanger, 4036 Norway

**Keywords:** Seaweed aquaculture, Rhodophyta, Bioactivity, Phenolics, Antioxidant peptide, Sustainability, Marine biology, Plant sciences, Chemical biology

## Abstract

*Palmaria palmata* is a highly nutritious seaweed and a prime candidate for developing sustainable aquaculture of human foodstuff in the North Atlantic, but it faces challenges due to high prices and quality inconsistencies. The present study evaluated the use of hydrogen peroxide (H_2_O_2_) treatment to consistently enhance the antioxidant capacity of this species. Medium-term (3–7 days), moderate (0.1-1 mM) treatment led to increased phenolic content and antioxidant activity, up to 2.2- and 5.4-fold baseline values, respectively. Proteomics analyses were performed to compare in vitro results to abundances of specific proteins. Increases in antioxidant power were tied to a decrease in growth-related proteins; but were not positively correlated to any specific protein group. These results suggest a medium-term, phenolic-driven response. Additional benefits of H_2_O_2_ treatment, including its potential for biofouling mitigation, may make this approach highly valuable for improving the quality and consistency of *P. palmata* and enhancing its valorisation as a functional food and commercial viability.

**Supplementary Information:**

The online version contains supplementary material available at 10.1038/s41598-025-03785-z.

## Introduction

The current food production industry is a major driver of climate change, while also being likely to face its severe effects. The need for novel, sustainably produced functional foods is rising due to increasing population levels and widespread malnutrition^1^. Seaweeds are nutritious, widespread and part of the traditional diets of many cultures globally. While traditionally wild-harvested, efficient methods for seaweed aquaculture have been developed over the past 70 years, particularly in East and South Asia^2,3^. These at-sea cultures require little resources and produce high yields, while contributing to climate change mitigation, water cleaning and coastal protection^4^. Recent developments for seaweed aquaculture in Western countries have largely focused on kelps, due to their cheap and simple cultivation. However, brown seaweeds are generally less nutritious than their green and red counterparts and have limited applications for human consumption^5^.

The red seaweed *Palmaria palmata* (known as dulse) is a medium-sized, intertidal macrophyte widely distributed in the North Atlantic. A traditional food in many North-Western European countries, it is valued for its high nutritional content and palatability. Proteins constitute up to 20% of the dry weight, roughly twice as much as commonly cultivated kelps^6,7^. *P. palmata* is also a valuable source of vitamins, including B12 – usually absent in plant foods – dietary fibres and minerals^8^.

As demand increases, wild stocks cannot be sustainably harvested. Despite growing interest, the development of aquaculture for this species is facing challenges^9^. Its complex heteromorphic life cycle is strictly seasonal and spread over two years, rendering the establishment of hatcheries onerous and costly^10^. It also grows to smaller sizes than kelps, thus reducing yields, and its nutritional properties can be variable^7^. While some of these obstacles will likely be removed as the industry grows, *P. palmata* will remain more expensive to cultivate than kelp species. Therefore, it is crucial to focus on the proper valorisation of this species as a high-quality functional food.

Bioactive properties and nutraceutical applications of *P. palmata* represent significant opportunities, as it has already documented antioxidant, anti-hypertensive and anti-inflammatory benefits^11^. Antioxidant foods can help reduce risks of oxidative stress-related illnesses, including cancer, heart disease or obesity, and are widely considered beneficial to health^12^. *P. palmata* is rich in polyphenols, important compounds for antioxidant bioactivity. Powerful antioxidant peptides have also been found in this species^13,14^. However, the antioxidant activity and protein and phenolic levels vary across seasons, populations and growing conditions^15^. FRAP values measured in different seasons decreased two-fold between April and October, and ORAC values decreased three-fold over the same period. While the peak of activity coincides with traditional harvest times, further cultivation development will require the development of practices including multiple harvests yearly. Therefore, methods to enhance the production of antioxidant compounds and ensure a consistent, high-quality functional food are essential to developing this species’ cultivation industry.

Hydrogen peroxide (H_2_O_2_) is a key signalling molecule in several pathways in higher plants, ranging from regulating photosynthesis and reproduction to triggering cell death in response to stress^16^. It is also strongly associated with oxidative stress metabolism and antioxidant response in seaweeds^17^. In *Ulva fasciata*, stress due to hypersalinity led to increases in H_2_O_2_ content as part of a response mitigating oxidative damage^18^. However, the potential for external H_2_O_2_ to trigger similar responses has largely been unexplored despite the potential production increase of valuable antioxidant compounds. This study aimed to investigate whether an external source of H_2_O_2_ could trigger defence metabolism in *P. palmata* without inducing excessive stress levels to boost the antioxidant properties of this seaweed. We hypothesised that (i) antioxidant activity can be increased by a medium-term treatment with H_2_O_2_; and (ii) changes in antioxidant activity may correlate with specific seaweed compounds.

## Materials and methods

### Algal material

Five vegetative sporophytes were sampled from a wild *P. palmata* population in Vistnes, Norway (58.983 ºN, 5.569 ºE) in November 2023. F/2 culture medium (Guillard, 1975; acquired from Varicon Aqua, Worcester, United Kingdom) based on sterile artificial seawater (ASW), with a salinity of 32 ± 1 ppm (Aquarium Systems, Sarrebourg, France), was used for all experiments. Sporophytes were cleaned, rinsed in ASW, placed in F/2 medium and acclimated at 10 ºC, 50 µmol photons m^− 2^ s^− 1^, 12 h:12 h light: dark (L: D) cycle for 48 h in a climate chamber (MLR-352-PE, PHCbi, Tokyo, Japan).

**Fig. 1:**
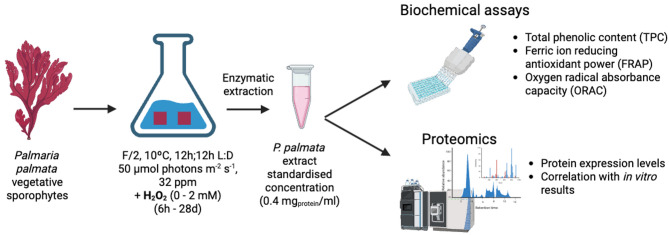
Experimental design. Image created using BioRender.

### Hydrogen peroxide treatment

Fragments averaging 4 cm^2^ were cut from young, pliable sporophyte thalli using a razor blade and dipped in clean ASW to remove any superficial fouling before transfer to 250 ml conical flasks containing 200 ml F/2 medium and H_2_O_2_ (0 – control, 0.1 mM, 0.5 mM. 1 mM, 2 mM, Fig. 1). These values were selected based on unpublished work attempting to use H_2_O_2_ baths for epiphyte control. Six fragments were added to each flask, and five replicates were used for each treatment, one per sporophyte collected (5 treatments * 5 replicates = 25 total flasks). From each sporophyte (mother plants), an additional fragment averaging 10 cm^2^ was cut and immediately frozen using liquid nitrogen and kept at -80 ºC to serve as T0 sample. Samples, kept in constant motion and aerated by bubbling, were maintained at 10 ⁰C, 50 µmol photons m^− 2^ s^− 1^, 12 h:12 h L: D cycle in a climate chamber (Fitotron SGC120, Weiss Technik, Reiskirchen, Germany) for the entirety of the experiment. The medium was changed weekly. At six time points during the experiment (6 h, 24 h, 3d, 7d, 14d, 28d), one of the fragments was removed from each flask, promptly frozen in liquid nitrogen and stored at -80 ºC. The time points were chosen to reflect shock response (6 h-3d^20^, short-term response (3-14d^21^ and medium-term response or adaptation (14-28d^22^. All fragments in 2 mM and 1 mM flasks had died after 3 and 14 days, respectively. Further results are not reported for 2 mM samples. After 21 and 28 days, biofouling and epiphyte growth was observed to be less present in fragments and flasks exposed to 0.1 and 0.5mM H_2_O_2_ compared to controls (Fig. S1).

### Seaweed extract Preparation and total soluble protein quantification

Cell contents extraction was realised according to Mæhre et al.^23^. Seaweed fragments were homogenised for 30 s in 28 µl/mg_seaweed_ of 0.05 M sodium acetate buffer (pH 5.0) and incubated for 30 min at 40 ⁰C with constant rotation. 2 µl/mg_seaweed_ of a solution containing 5 U/ml of cellulase and xylanase was added, and the slurries were incubated for 18 h at 40 ⁰C with constant rotation. Samples were centrifuged at 4000 x *g* for 15 min at 4 ⁰C. Supernatants were transferred to new tubes, and pellets re-suspended in 8 µl/mg seaweed of 0.1 M NaOH and incubated for 24 h at 23 ⁰C with constant rotation. Samples were further centrifuged at 4000 x *g* for 15 min at 4 ⁰C. The supernatants were removed, combined with the previous supernatants, and the protein concentrations of the resulting extracts were determined using the Pierce-modified Lowry protein assay kit (Thermo-Fischer Scientific, Waltham, USA; catalogue nº23,240).

Samples were diluted to a concentration of 0.4 mg_protein_/ml for the in vitro assays (Sect. 2.4).

### *In vitro* assays

The total phenolic content (TPC) of seaweed extracts was measured according toWang et al.^24^, with volumes divided 1:50 for use in 96-well plates, and absorbance read at 750 nm. All spectrophotometric and fluorometric measurements in this study were taken using a SpectraMax iD5 Multi-Mode Microplate Reader (Molecular Devices, San Jose, USA).

The ferric ion reducing antioxidant power (FRAP) assay is sensitive to polyphenol-related antioxidant activity and was performed to assess non-oxygen-radical antioxidant power.

The oxygen radical absorbance capacity (ORAC) assay was performed to measure the samples’ ability to neutralise reactive oxygen species (ROS). Both FRAP and ORAC assays were conducted as previously described in Liboureau & Pampanin^14^.

### Protein identification and quantification

Samples from all experimental groups at time points 24 h, 3 and 7 days were selected for LC-MS/MS analysis according to their in vitro bioactivity. For each treatment, seaweed extracts from all five replicates were pooled for analysis (3 time points × 4 treatments = 12 samples in total). Protein identification and quantification were carried out according to the method previously described in Liboureau & Pampanin^14^ (SM1). Peptides detected in LC-MS/MS were searched against the NCBI Rhodophyta database (downloaded July 2024; 459,900 sequences), and protein abundances were calculated as the sum of abundances of peptides mapped to each protein.

### Statistical analysis

Statistical analyses were conducted using the R statistical environment^25^ and the PERMANOVA module of Primer 6^26,27^. In vitro data was tested for normality and homoscedasticity using the Shapiro-Wilk and Levene’s tests, respectively. ANOVAs were performed where assumptions were met, and PERMANOVA otherwise.

The total soluble protein content, ORAC (square-root transformed) and FRAP results were assessed using a two-factor ANOVA (fixed factors: treatment, time; *n* = 5), and post-hoc Tukey tests. TPC was analysed using a two-factor PERMANOVA (fixed factors: treatment, time; *n* = 5) and post-hoc pairwise t-tests, both using Euclidean distances and 9999 permutations. Pearson correlation coefficients were calculated between all four in vitro results, and p-values adjusted using Bonferroni corrections for multiple testing.

For in silico protein identification results, correlation analyses were performed between the abundances of each protein and in vitro results, using the Kendall rank correlation coefficient.

Principal component analysis was performed using the factoextra and FactoMineR packages in R^28,29^. Protein groups with 3 or more proteins were analysed using pairwise t-tests between all samples and using values from different proteins as virtual replicates.

Results were considered significant for *p* < 0.05.

## Results and discussion

### Total soluble protein content

**Fig. 2:**
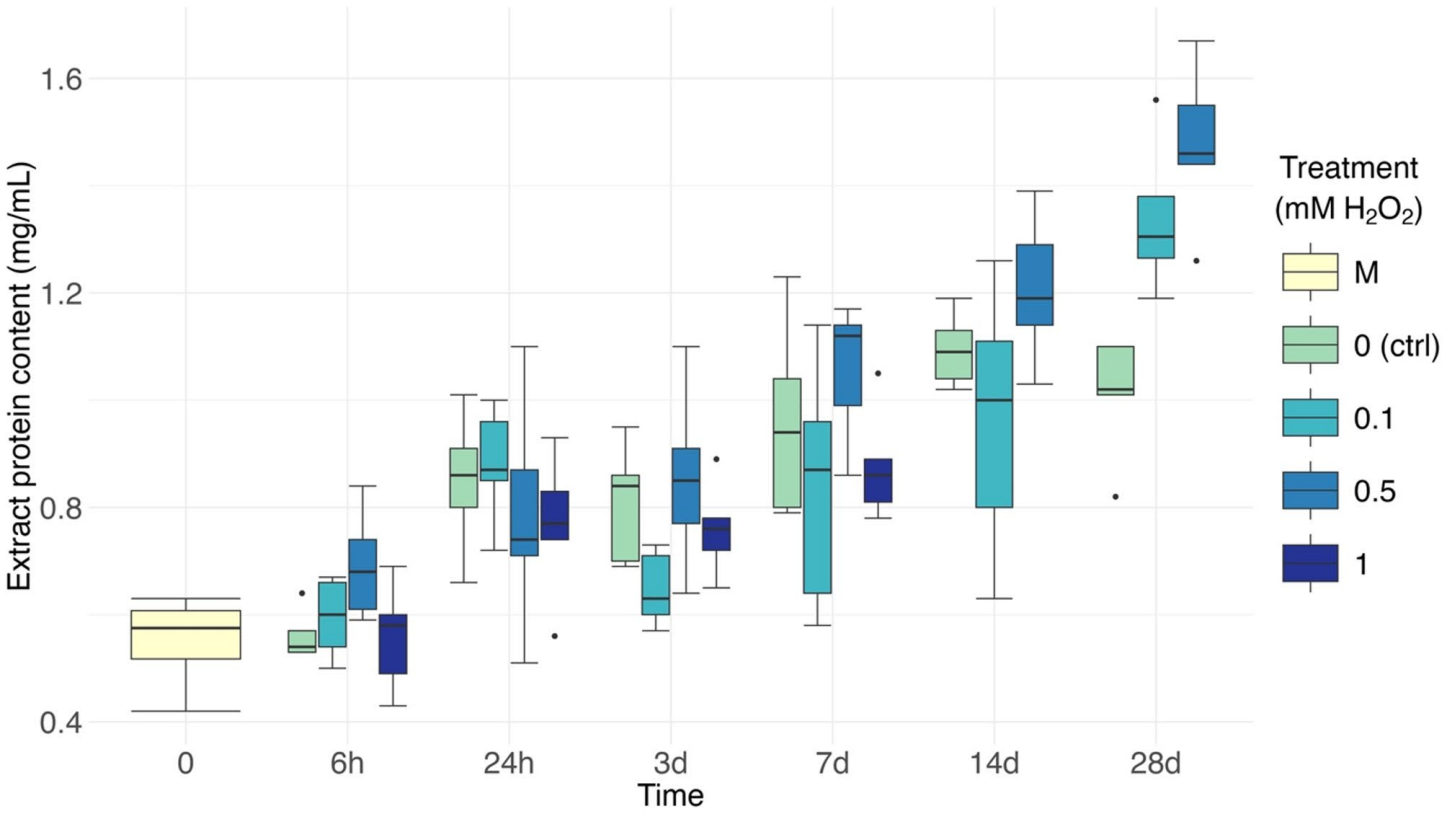
Total protein content of seaweed extract from fragments of *Palmaria palmata* exposed to hydrogen peroxide treatment over 28 days. Data reported as mg_protein_/ml_extract_. Boxplots with median, boxes for 25th and 75th percentiles and dots indicating outliers (*n* = 5). M = mother plants (representing T0).

The treatment × time interaction had a significant effect on the total soluble protein content of the seaweed (table S1). For all experimental groups, except 1mM, the protein content increased over time (Fig. 2, S2). Control fragments had significantly higher protein concentrations after 7–28 days (1.02 mg_protein_/ml_extract_ on average) compared to the mother plants and the control group after 6 h (0.56 mg/ml, 1.82-fold increase). Fragments exposed to 0.1 mM H_2_O_2_ had more protein after 28 days (1.34 mg/ml) than at all other time points (0.784 mg/ml, 1.71-fold increase). Exposed to 0.5 mM H_2_O_2_, fragments had significantly more protein than mother plants (0.55 mg/ml) from 7 days onward (1.25 mg/ml, 2.27-fold increase), and after 28 days had the highest protein content of all samples (1.48 mg/ml). The high nutrient availability in the cultivation medium is most likely responsible for such increases observed in both control and treated groups. Similar increases in protein contents have been reported during the initial weeks of in-lab cultivation with added nutrients^30,31^. Overall, the wide variation (0.55–1.34 mg/ml) illustrates the importance of developing cultivation methods designed to maintain high protein contents and ensure the consistency and quality of products.

Across treatments, the only significant difference was recorded at 28 days, when fragments exposed to 0.5 mM H_2_O_2_ had 1.47-fold more protein than control fragments (1.01 mg/ml). The effects of H_2_O_2_ treatment on total protein content seem limited. A further study may help indicate whether the increase observed after 28 days is maintained over time. From a commercial perspective, such a prolonged treatment would require land-based facilities to allow for complete control over the seaweed conditions prior to harvest. While this could lead to improvements in the quality and consistency of the product^32^, such facilities are expensive and would require a strong valorisation of the end product to be commercially viable^33^. Cultivation of *P. palmata* as a health food for human consumption is unlikely to be viable in this context, and the development of methods to optimise protein content during at-sea cultivation should be prioritised.

### *In vitro* assays

#### Total phenolic content

**Fig. 3:**
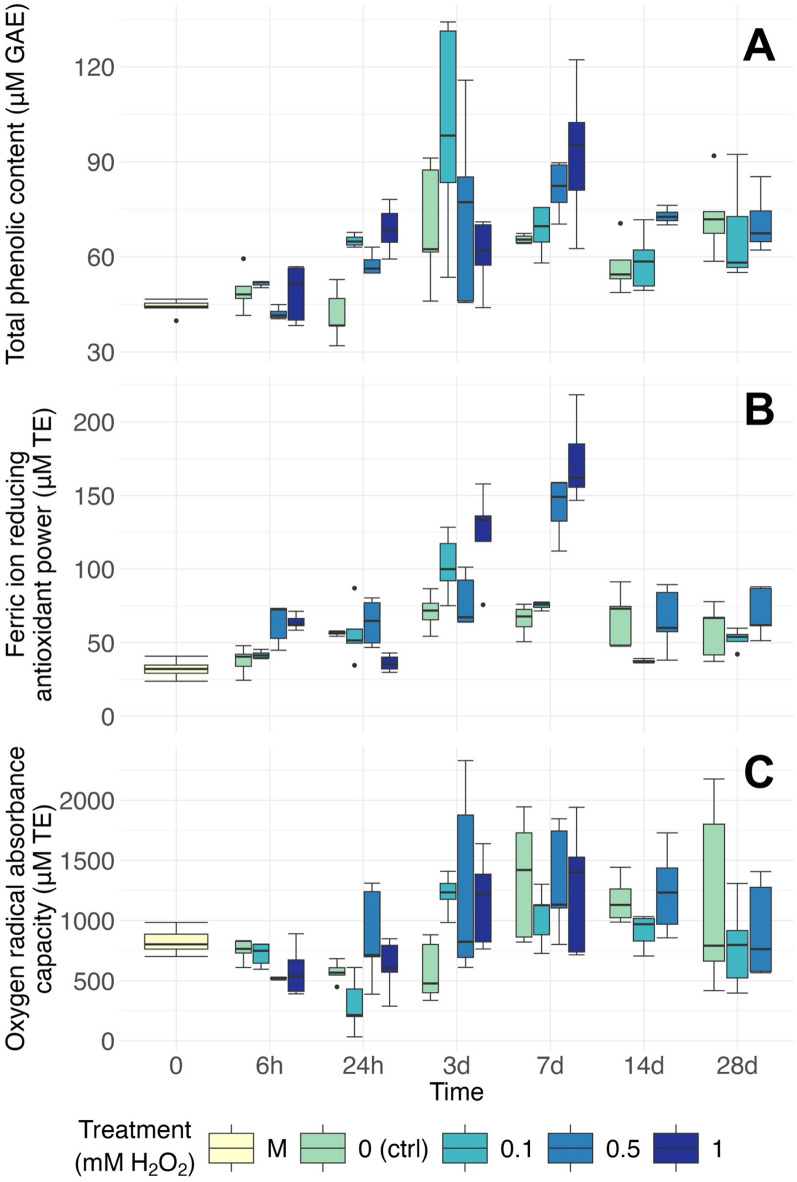
Antioxidant activity of seaweed extracts from *Palmaria palmata* exposed to hydrogen peroxide treatment over 28 days. (**A**): Total phenolic content of seaweed extracts. Data reported as µM of gallic acid equivalent. (**B**): Ferric ion reducing antioxidant power. (**C**): Oxygen radical absorbance capacity. (**B**) and (**C**): data reported as µM Trolox equivalent. Boxplots with median, boxes for 25th and 75th percentiles and dots indicating outliers (*n* = 5). M = mother plants (representing T0).

The treatment × time interaction significantly affected TPC in the samples (Table S2). Control fragments had significantly higher TPC than mother plants after 3 days and all further time points, while all test treatments had higher TPC than mother plants from 24 h onwards (Fig. 3A, S3A). This similarity with protein content contradicts the protein competition model, which predicts that protein production is favoured over that of phenolic compounds when high nitrogen levels are available^34,35^. Increased nitrate availability was not reflected in phenolic content in *Porphyra linearis*, but did increase the total protein content^36^. In *P. palmata*, however, phenolic content was correlated to total soluble protein in a nutrient enrichment experiment^30^, suggesting a potentially species-specific nutrient allocation. The nutrient excess afforded by the F/2 medium in both *P. palmata* experiments may also have afforded sufficient phenylalanine for high production of both protein and phenolics.

For all test treatments, TPC appeared to increase over the early stages of the experiment, peaking after 3 (0.1 mM: 100.16 µM GAE) or 7 (0.5 mM: 81.74 µM GAE; 1mM: 92.74 µM GAE) days of treatment, before decreasing slightly and stabilising (Fig. 3A). Fragments exposed to 0.5 mM H_2_O_2_ had significantly higher TPC than control fragments at 24 h and 7 d. Samples exposed to 1mM displayed higher TPC than both control and ones exposed to 0.5 mM after 24 h. These results support the hypothesis that the H_2_O_2_ treatment would trigger an increase in antioxidant compound production. This response was strong but relatively short-lived, and higher concentrations of H_2_O_2_ rapidly killed the fragments. This is likely a defence response to oxidative stress caused by the treatments. Phenolic compounds are often expressed in response to stress in seaweed and have been suggested as potential ecological biomarkers in wild populations^37^. Increases in the production of phenolic acids were also noted in microalgae exposed to oxidative stress from heavy metals for 48 h-72 h^38^. In grapes, oxidative stress led to increased concentrations of several phenolic compounds after 12–48 h of exposure to menadione^39^. Flavonoids, a class of polyphenols, were also found to help Mediterranean plants endure environmental oxidative stress^40^. This may be reflected in the *P. palmata* fragments exposed to 0.5 and 1 mM H_2_O_2_, where TPC increased following treatment.

Polyphenols are naturally present in all plants and provide many health benefits when consumed, including antioxidant, anti-inflammatory and antibacterial properties^41^. In general, seaweeds tend to have high concentrations of polyphenols^42^. However, they are often described as “total phenolics”, and further research into the specific compounds and their properties is essential to better understand their functions and potential benefits^11^. Further, the bioaccessibility and bioavailability of phenolic compounds in unprocessed seaweed are relatively low, and proper processing methods need to be developed to take full advantage of potential health benefits^43^. Polyphenols are important in valorising *P. palmata* as food for human consumption, and these results suggest the possibility of inducing concentration increases; further studies will help elucidate their nature, value and accessibility.

#### Ferric ion reducing antioxidant power

FRAP results showed similar patterns to TPC results (Fig. 3B and A, respectively). The treatment × time interaction had a significant effect on FRAP values (Table S3). All the samples treated with H_2_O_2_ had significantly higher FRAP values after 3 and 7 days of exposure compared to the mother plants. Samples exposed to 0.1 mM H_2_O_2_ peaked after 3 days (102.6 µM TE), significantly higher than at all other time points except 7d (47.2 µM TE, 2.17-fold increase). In samples exposed to 0.5mM and 1mM, FRAP value maxima were observed after 7 days (142.2 and 173.5 µM TE, resp.), significantly higher than at all other time points (68.2 and 74.8 µM TE; 2.09 and 2.32-fold higher, resp.). At 3 days, FRAP was significantly higher in samples exposed to 1mM than in samples subjected to control and 0.5mM treatments. After 7 days, fragments exposed to both 0.5 mM and 1 mM had significantly higher FRAP activity than controls and those exposed to 0.1 mM of H_2_O_2_. Mother plants had FRAP activity (32.146 µM TE) comparable with published results^14,15^. The H_2_O_2_ treatment led to large increases at all concentrations and up to 5.4-fold in fragments exposed to 1mM for 7 days, demonstrating its strong potential in promoting the production of antioxidant compounds in *P. palmata*.

#### Oxygen radical absorbance capacity

ORAC results showed a wide variation, ranging from 298 µM TE (recorded at 0.1 mM H_2_O_2_ exposure for 24 h) to 1356 µM (in the control group after 7d) (Fig. 3C). However, significant variations were also observed within experimental groups, and no clear patterns can be observed from the data. The treatment × time interaction was significant (Table S4), but the only significant differences were in fragments exposed to 0.1 mM H_2_O_2_, where ORAC values were lower after 24 h compared to 3 and 7 days. This difference appears to be due to a short-term, unexplained decrease in ORAC values at 24 h rather than to treatment effects. While values appear to increase after 3–14 days, with some samples displaying very high ORAC (max = 2329 µM TE in a sample exposed to 0.5mM for 3 days), the wide variation within groups suggests that responses to H_2_O_2_ treatment in ORAC, if any, are strongly affected by individual variation. Additionally, plants are known to produce H_2_O_2_, itself a ROS producer, as a response to stress in sufficient levels for it to be suggested as a potential bioindicator^17^. It is possible that the presence of H_2_O_2_-generated ROS from both treatments and plants increased the variability in ORAC values.

#### In vitro assays correlations

Pearson correlation coefficients between in vitro assay results showed a weak correlation between protein content and ORAC values (0.270, Table 1) and a moderate correlation between FRAP and TPC (0.514) and FRAP and ORAC (0.394), with all other correlations not statistically significant. The correlation between FRAP and TPC is unsurprising, considering the former was selected for its sensitivity to phenolic compounds. FRAP measures the general reducing ability of the extracts, while ORAC specifically tests the scavenging of peroxyl radicals through hydrogen atom donation, and discrepancies between the two are common^44,45^. The lack of correlation between TPC and ORAC may suggest that the phenolic compounds secreted in response to H_2_O_2_ treatment favour electron donation as a reducing mechanism. Aromatic rings in polyphenols can stabilise radicals following electron donation, making them consistently strong reducing agents^46^. This mechanism is favoured over hydrogen atom transfer in some phenolic compounds, such as flavonoids^47^. The latter are known to be involved in rapid response and oxidative stress mitigation in plants^48,49^ and may drive, in part, the discrepancy between TPC-FRAP and TPC-ORAC correlations. The correlation between protein content and ORAC results may also indicate a role of proteins in antioxidant defence mechanisms, especially after the initial peak in phenolic content and FRAP activity. These results suggest a complex response to H_2_O_2_ exposure involving several compounds to alleviate the resulting stress over time and with various mechanisms. They also highlight the importance of using several antioxidant assays to obtain results with potential relevance to in vivo response^50^.

**Table 1:** Pearson correlation coefficients between measured in vitro properties of *P. palmata* fragments exposed to hydrogen peroxide. Statistically significant correlations are highlighted in bold (p-adjusted < 0.05). *TPC: * total phenolic content, *FRAP: * ferric ion reducing antioxidant power,* ORAC:*  oxygen radical absorbance capacity.

	Protein content	TPC	FRAP	ORAC
Protein content	1	0.214	0.115	**0.270**
TPC	0.214	1	**0.514**	0.224
FRAP	0.115	**0.514**	1	**0.394**
ORAC	**0.270**	0.224	**0.394**	1

### Protein identification and quantification

A total of 743 proteins were identified from 2,068 peptides. The most represented protein families were RuBisCO (71 proteins), pigments phycoerythrin (24) and allophycocyanin (17), ATP synthases (16), photosystems I and II (13 and 12, resp.) and phycobilisomes (10). Many unnamed (189) and hypothetical (57) proteins were also identified.

A significant correlation was found between the FRAP assay data and the abundances of 223 proteins, of which 132 were correlated positively and 91 negatively. ORAC results were positively correlated with 71 proteins and negatively with 79 (150 proteins in total). TPC values were positively correlated with 53 proteins and negatively with 12. Many protein groups related to photosynthesis, basic metabolism and growth were negatively correlated with results from both antioxidant assays (Table 2), suggesting a downregulation due to the treatment. Adaptive trade-offs in resource allocation between growth and defence are known to exist in plants^51,52^. These results appear to support the existence of similar processes in *P. palmata*. The positive correlation between TPC and ribosomal protein abundance may be explained by the key role of ribosome binding sites in the production of phenolic compounds^53,54^, thus leading to upregulation after moderate H_2_O_2_ exposure.

**Table 2:** Protein groups with at least three individual proteins having abundances significantly correlated with in vitro assays. The number of proteins significantly correlated with each assay is reported in brackets.

Assay	Positive correlation	Negative correlation
FRAP	Unnamed/hypothetical (70) Elongation factor (4)	Unnamed/hypothetical (21) Photosystem proteins (19) Pigments (16) RuBisCO (7) ATP synthases (5)
ORAC	Unnamed/hypothetical (36)	Unnamed/hypothetical (16) Pigments (12) Photosystem proteins (14) RuBisCO (9) ATP synthases (7)
TPC	Unnamed/hypothetical (27) Ribosomal proteins (15)	

Pairwise t-tests between protein groups showed significant differences between samples in only two protein groups, RNA polymerases and protochlorophyllide reductases, with both showing similar patterns (Table 3). Abundances of these proteins increased between 1 and 7 days in fragments exposed to 0.1 and 0.5 mM H_2_O_2_ but were lower at 7 days in samples exposed to 1 mM than in all other samples. Both RNA polymerase II and protochlorophyllide reductase are known to be transiently less expressed in plants exposed to stress^55,56^. The latter is involved in chlorophyll biosynthesis and has been used as a proxy measurement for organism health^57^. The present results may show the gradual recovery in photosynthetic function between days 1 and 7 following initial shock in samples exposed to 0.1 and 0.5 mM H_2_O_2_ and illustrate the poor physiological health of fragments exposed to 1 mM H_2_O_2_ after 7 days.

**Table 3:** Protein groups presenting significant differences in abundances between samples of *Palmaria palmata* exposed to oxidative stress.

Protein group	Significant pairwise t-test results
RNA polymerases	0.1mM 1d < 0.1mM 3d < 0.1mM 7d
	0.5mM 1d < 0.5mM 3d & 7d
	1mM 7d < C, 0.1 & 0.5mM 7d
Protochlorophyllide reductases	0.1mM 1d < 0.1mM 7d
	0.5mM 1d < 0.5mM 7d
	1mM 7d < C, 0.1 & 0.5mM 7d
	1mM 1d < C 1d

The principal component analysis did not show differences between the experimental groups, independently of the dose and the time (Fig. S4). The sample treated with 1 mM H_2_O_2_ for 7 days was the only one which separated from the rest of the groups. This is clearly explained by the poor physiological health of the fragments, which resulted in an expected death before the end of the experiment.

Overall, the proteomic analyses highlight the need to dedicate more research and resources to developing modern, omics-based methods for algal and seaweed science. In all bioassays, the protein groups with the highest numbers of correlated proteins were unnamed or hypothetical proteins (Table 2), highlighting the need to obtain and annotate proteomes for various seaweed species, including *P. palmata*. Similarly, attempts at metabolic pathway analyses were unsuccessful due to the absence or severe lack of appropriate data. Proprietary software, such as Ingenuity Pathway Analysis (QIAGEN Inc., https://digitalinsights.qiagen.com/IPA), did not include any support for plant research. Open-source databases such as the KEGG pathway database^58^ or The Arabidopsis Information Resource^59^ (TAIR) did include pathways about model higher plants, but the lack of algae-specific pathways or confirmed orthologs between algal species and model organisms prevented further progress. If seaweed aquaculture is to become a serious, sustainable competitor to land-based agriculture, such resources must urgently be developed and their use standardised in relevant research.

### Perspectives for commercial aquaculture

Medium-term H_2_O_2_ treatment was successful in triggering increases in antioxidant activity in *P. palmata*, which appear to be mainly related to the induced production of phenolic compounds. This species is complex and presents several challenges for aquaculture development. Increased valorisation opportunities, as presented in this study, can help balance the increased costs and improve commercial prospects for this seaweed. However, the effective range of H_2_O_2_ concentrations must be refined and adapted to commercial conditions to produce desired effects (> 0.1 mM in this study) without killing the plants (< 1 mM here). Similarly, exposure duration must be calibrated to find a balance between obtaining desired effects and minimising costs of keeping plants in land facilities for treatment. Developing monitoring methods to reliably process and stabilise biomass during the peak of antioxidant activity would help improve consistency in the product quality.

H_2_O_2_ treatment may provide additional benefits for aquaculture. It can be used to treat epiphytic biofouling, an important issue in seaweed aquaculture which reduces the yields and value of products and is currently lacking reliable solutions^60,61^. In this study, flasks and fragments exposed to higher, non-lethal H_2_O_2_ concentrations showed reduced biofouling, especially in the later stages of the experiment (Fig. S1). H_2_O_2_ is also increasingly used as a disinfectant in the food industry and has been shown to cause little to no damage to product quality^62,63^. While current methods generally use short treatments in concentrated H_2_O_2_ solutions, the results presented suggest that longer, more gentle treatments could also be successful in improving the nutritional and nutraceutical value of *P. palmata* while increasing product quality and safety.

## Conclusion

Our results confirm the possibility of using H_2_O_2_ to increase the antioxidant activity of *Palmaria palmata* by promoting the production of specific compound groups. The treatment increased phenolic contents and FRAP antioxidant activity in the medium term, although levels decreased after 7–14 days. Therefore, the effects are likely associated with a transient stress response, prior to longer-term adaptation mechanisms. Proteomics analyses showed that many protein groups associated with growth were negatively correlated with measured antioxidant activities, suggesting a trade-off between defence and growth.

The chosen highest H_2_O_2_ concentrations were lethal, highlighting the potential to overwhelm the individuals’ response and the need to develop specific methods for using such treatments in commercial contexts. Available omics data and tools were insufficient to identify specific pathways related to the treatment effects, and we urge the widespread development and use of advanced, modern research methods to support the development of a sustainable seaweed aquaculture industry.

Overall, this study offers a simple approach with multiple potential benefits in cultivating *P. palmata.* It shows the potential to improve the quality and consistency of this and potentially other seaweeds, to valorise and promote their development as sustainable, functional foods with wide applications.

## Electronic supplementary material

Below is the link to the electronic supplementary material.

Supplementary Material 1

## Data Availability

All experimental data and code for analyses is available on Mendeley Data - Liboureau, Pierre; Pampanin, Daniela Maria (2025), “Hydrogen peroxide treatment data”, Mendeley Data, V4, doi: 10.17632/x578jn49pb.4.
