# Supplementary material for: Boosting the antioxidant power of Palmaria palmata using hydrogen peroxide
Source: Sci Rep. 2025 Jun 5;15:19834. doi: 10.1038/s41598-025-03785-z (PMC12141464; doi:10.1038/s41598-025-03785-z)
Supplement: Supplementary file 1 — Supplementary Material 1 [file 41598_2025_3785_MOESM1_ESM.docx]

**Effects of hydrogen peroxide treatment on the antioxidant power of *Palmaria palmata***

**SUPPLEMENTARY MATERIAL**

**Supplementary methods 1: Protein identification and quantification**

Samples were lysed with 20% sodium dodecyl sulfate (SDS), reduced in dithiothreitol and alkylated with indole-3-acetic acid, and digested using the single-pot, solid-phase-enhanced sample preparation method (Hughes et al., 2019). Digestion was performed using a 1:1 ratio of magnetic carboxylate-modified beads (GE Healthcare, Chicago, IL, USA). A bead solution at 100 µg µl^-1^ was added to each replicate to a bead:protein ratio of 10:1 (*w*/*w*). Proteins solubilised in SDS were washed twice with 80% (*v*:*v*) ethanol to remove possible traces of SDS.

*2.7.1 NanoLC-Orbitrap Eclipse mass spectrometry w/FAIMS pro interphase*

About 0.5 µg protein as tryptic peptides dissolved in 2% acetonitrile (ACN), 0.5% formic acid, were injected into a low-flow liquid chromatography system (Ultimate 3000 RSLC system, Thermo Scientific, Sunnyvale, California, USA) connected online to a Orbitrap Eclipse mass spectrometer (Thermo Scientific, Bremen, Germany) equipped with EASY-spray nano-electrospray ion source (Thermo Scientific). Samples were loaded and desalted on a pre-column (Acclaim PepMap 100, 2 cm x 75 µm ID nanoViper column, packed with 3 µm C18 beads) at a flow rate of 5 µl min^-1^ for 5 min with 0.1% trifluoroacetic acid (TFA). Peptides were separated during a biphasic ACN gradient from two nanoflow ultra-performance liquid chromatography pumps (flow rate of 200 nl min^-1^) on a 50 cm analytical column (PepMap RSLC, 50 cm x 75 µm ID EASY-spray column, packed with 2 µm C18 beads). Solvents A and B were 0.1% TFA (v/v) in water and 100% ACN respectively. The gradient composition was 5% B during trapping (5 min) followed by 5-8% B over 1 min, 8–25% B for the next 124 min, 25-36% B over 30 min, and 36-80% B over 5 min. Elution of very hydrophobic peptides and conditioning of the column were performed during 10 min isocratic elution with 80% B and 15 min isocratic conditioning with 5% B. Instrument control was through Thermo Scientific SII for Xcalibur 1.6.

*2.7.2 Data analysis*

Sequest HT database search engine (Eng et al., 1994), with Percolator validation (Käll et al., 2008; FDR < 0.01), was used for searching the raw files in Proteome Discoverer software v2.5 (Thermo Fisher Scientific). The search was conducted against the NCBI Rhodophyta database (downloaded July 2024; 459900 sequences). The default settings were applied, and normalisation was performed using the sum of all peptide amounts. Protein abundances were calculated by summing sample abundances of the connected peptide groups. The software performed protein grouping, and reported results were filtered for master proteins.

Eng, J. K., McCormack, A. L., & Yates, J. R. (1994). An approach to correlate tandem mass spectral data of peptides with amino acid sequences in a protein database. *Journal of the American Society for Mass Spectrometry*, *5*(11), 976–989. https://doi.org/10.1016/1044-0305(94)80016-2

Hughes, C. S., Moggridge, S., Müller, T., Sorensen, P. H., Morin, G. B., & Krijgsveld, J. (2019). Single-pot, solid-phase-enhanced sample preparation for proteomics experiments. *Nature Protocols*, *14*(1), Article 1. https://doi.org/10.1038/s41596-018-0082-x

Käll, L., Storey, J. D., MacCoss, M. J., & Noble, W. S. (2008). Assigning Significance to Peptides Identified by Tandem Mass Spectrometry Using Decoy Databases. *Journal of Proteome Research*, *7*(1), 29–34. https://doi.org/10.1021/pr700600n

**
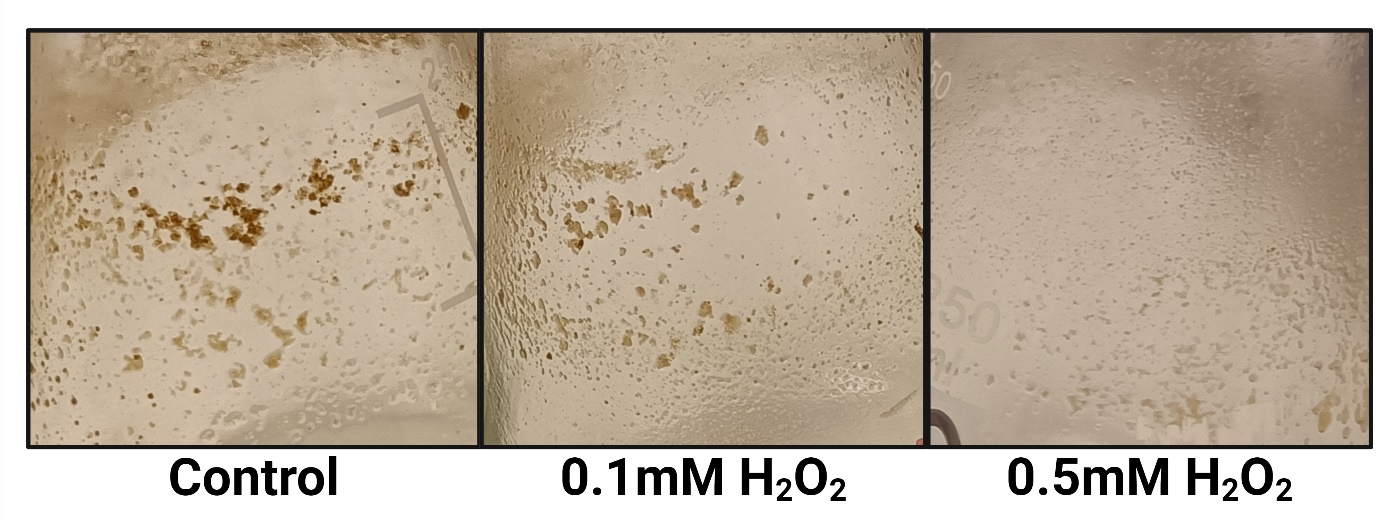
**

Figure S1: Biofouling on culture flasks after 28 days of Palmaria palmata culture with hydrogen peroxide concentrations relevant for promoting antioxidant power.


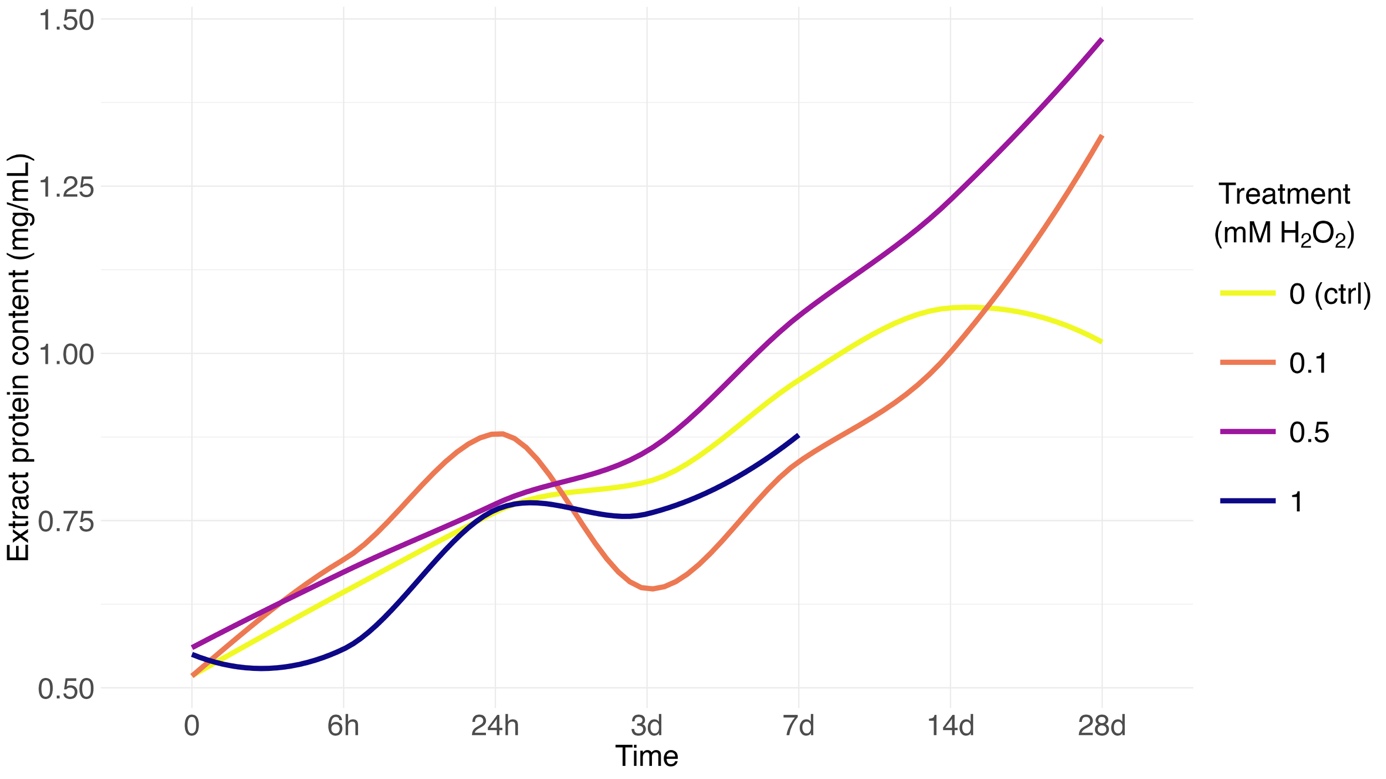


Figure S2: Total protein content of seaweed extract *from fragments of* Palmaria palmata *exposed to hydrogen peroxide treatment* over 28 days. Data reported as mg protein/ml extract. Conditional means with locally estimated scatterplot smoothing (LOESS, n = 5).


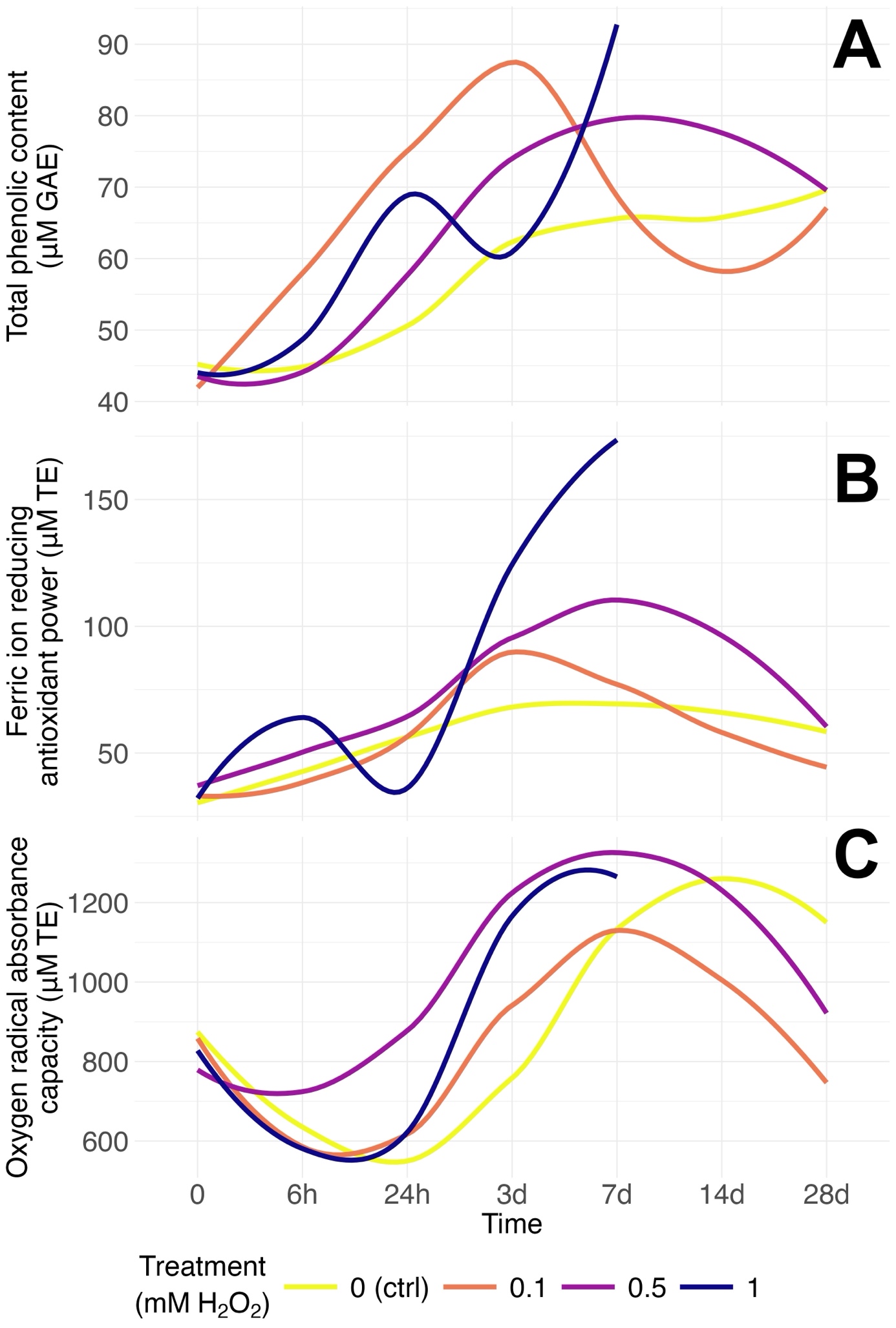


Figure S3: Figure 3: Antioxidant activity of seaweed extracts from Palmaria palmata exposed to hydrogen peroxide treatment over 28 days. **A:** Total phenolic content of seaweed extracts. Data reported as μM of gallic acid equivalent. **B:** Ferric ion reducing antioxidant power. **C:** Oxygen radical absorbance capacity. B and C: data reported as μM Trolox equivalent. Conditional means with locally estimated scatterplot smoothing (LOESS, n = 5).


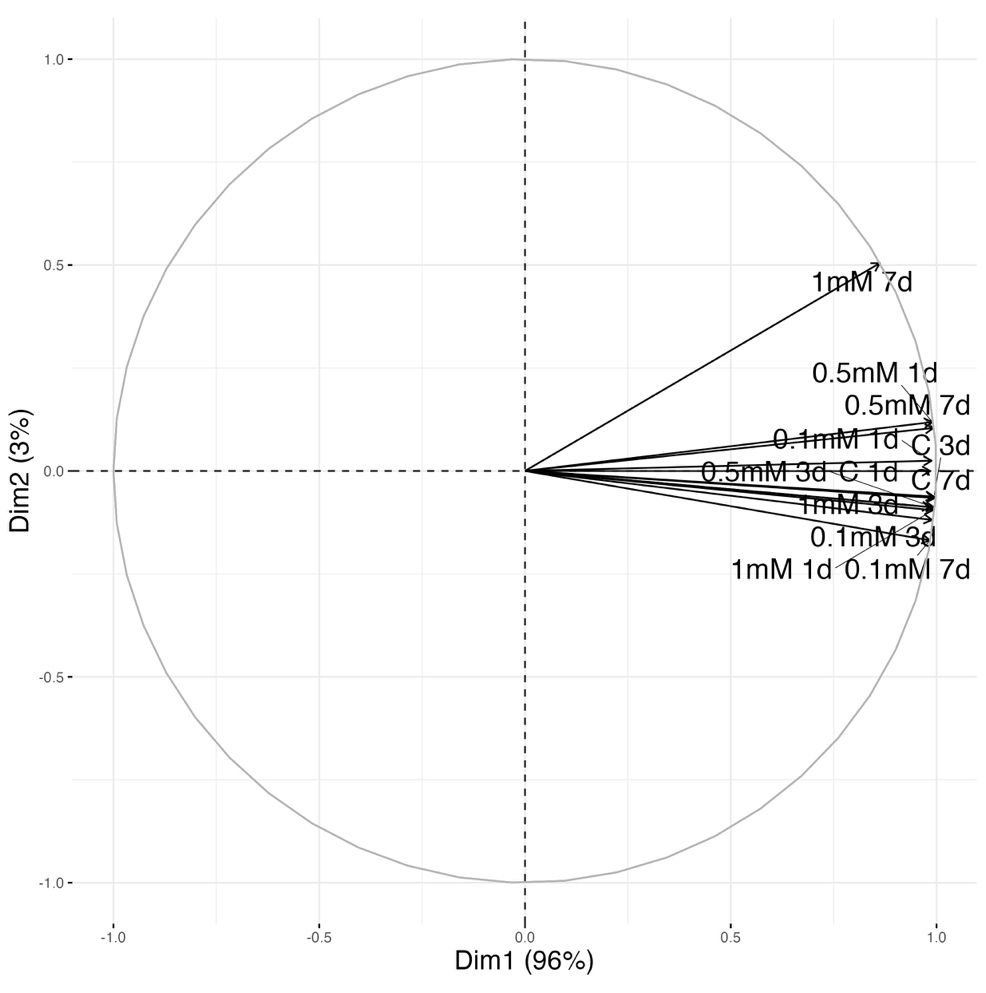


Figure S4: Principal component analysis of samples of Palmaria palmata exposed to oxidative stress, using abundances of identified proteins as variables. The first part of labels represents H_2_O_2_ concentrations (C = control) and the second the time point (d = days).

Table S1: ANOVA for the effects of treatment and time on the total soluble protein content of *Palmaria palmata*. Significant interactions or main effects are highlighted in bold.

| Factor | df | SS | MS | F | p |
| --- | --- | --- | --- | --- | --- |
| Treatment | 4 | 1.354 | 0.339 | 17.371 | **< 0.001** |
| Time | 5 | 4.200 | 0.840 | 43.109 | **< 0.001** |
| Treatment × Time | 13 | 0.673 | 0.052 | 2.657 | **0.003** |
| Residuals | 90 | 1.754 | 0.020 |  |  |
|  |  |  |  |  |  |

Table S2: PERMANOVA for the effects of treatment and time on the total phenolic content of extracts of *Palmaria palmata* with uniform protein concentration. Significant interactions or main effects are highlighted in bold.

| Factor | df | SS | MS | Pseudo-F | P(perm) |
| --- | --- | --- | --- | --- | --- |
| Treatment | 4 | 1073.2 | 357.75 | 1.872 | 0.139 |
| Time | 5 | 12477 | 2495.5 | 13.057 | **< 0.001** |
| Treatment × Time | 13 | 8109.6 | 623.82 | 3.264 | **< 0.001** |
| Residuals | 83 | 15863 | 191.12 |  |  |

Table S3: ANOVA for the effects of treatment and time on the ferric ion reducing antioxidant power (FRAP) of extracts of *Palmaria palmata* with uniform protein concentration. Significant interactions or main effects are highlighted in bold.

| Factor | df | SS | MS | F | p |
| --- | --- | --- | --- | --- | --- |
| Treatment | 4 | 32719 | 8178 | 32.03 | **< 0.001** |
| Time | 5 | 62379 | 12476 | 48.87 | **< 0.001** |
| Treatment × Time | 13 | 35542 | 2734 | 10.71 | **< 0.001** |
| Residuals | 87 | 22209 | 255 |  |  |

Table S4: ANOVA for the effects of treatment and time on the oxygen radical absorbance capacity of extracts of *Palmaria palmata*. Statistical differences are highlighted in bold.

| Factor | df | SS | MS | F | p |
| --- | --- | --- | --- | --- | --- |
| Treatment | 4 | 218.1 | 54.5 | 1.664 | 0.165 |
| Time | 5 | 1873.2 | 374.6 | 11.435 | **< 0.001** |
| Treatment × Time | 13 | 930.2 | 71.6 | 2.184 | **0.016** |
| Residuals | 90 | 2948.7 | 32.8 |  |  |
